# Machine Learning-Driven Radiomics Analysis for Distinguishing Mucinous and Non-Mucinous Pancreatic Cystic Lesions: A Multicentric Study

**DOI:** 10.3390/jimaging11030068

**Published:** 2025-02-20

**Authors:** Neus Torra-Ferrer, Maria Montserrat Duh, Queralt Grau-Ortega, Daniel Cañadas-Gómez, Juan Moreno-Vedia, Meritxell Riera-Marín, Melanie Aliaga-Lavrijsen, Mateu Serra-Prat, Javier García López, Miguel Ángel González-Ballester, Maria Teresa Fernández-Planas, Júlia Rodríguez-Comas

**Affiliations:** 1 Department of Radiology, Hospital of Mataró (Consorci Sanitari del Maresme), C/ Cirera 230, 08304 Mataró, Spain; ntorra@csdm.cat (N.T.-F.); mduh@csdm.cat (M.M.D.); mfernandezpl@csdm.cat (M.T.F.-P.); 2 Department of Radiology, Hospital Universitari de Girona Josep Trueta, Avinguda de França, S/N, 17007 Girona, Spain; queraltgrau@gmail.com; 3 Scientific and Technical Department, Sycai Technologies S.L., C/ Llacuna 162, 2nd Floor, 08018 Barcelona, Spain; dcanadas@sycaitechnologies.com (D.C.-G.); jmoreno@sycaitechnologies.com (J.M.-V.); mriera@sycaitechnologies.com (M.R.-M.); melaniealiaga@gmail.com (M.A.-L.); j.garcia@sycaitechnologies.com (J.G.L.); 4 Research Unit, Hospital de Mataró (Consorci Sanitari del Maresme), C/ Cirera 230, 08304 Mataró, Spain; mserra@csdm.cat; 5 BCN MedTech, Universitat Pompeu Fabra (UPF), Edificio Tànger (Campus de Comunicació Poblenou), C/ Tànger 122-140, 08018 Barcelona, Spain; ma.gonzalez@upf.edu; 6 Catalan Institution for Research and Advanced Studies (ICREA), Passeig Lluis Companys 23, 08010 Barcelona, Spain

**Keywords:** pancreatic cystic lesions, radiomics, feature extraction, image processing, deep features, computer vision, machine learning

## Abstract

The increasing use of high-resolution cross-sectional imaging has significantly enhanced the detection of pancreatic cystic lesions (PCLs), including pseudocysts and neoplastic entities such as IPMN, MCN, and SCN. However, accurate categorization of PCLs remains a challenge. This study aims to improve PCL evaluation by developing and validating a radiomics-based software tool leveraging machine learning (ML) for lesion classification. The model categorizes PCLs into mucinous and non-mucinous types using a custom dataset of 261 CT examinations, with 156 images for training and 105 for external validation. Three experienced radiologists manually delineated the images, extracting 38 radiological and 214 radiomic features using the Pyradiomics module in Python 3.13.2. Feature selection was performed using Least Absolute Shrinkage and Selection Operator (LASSO) regression, followed by classification with an Adaptive Boosting (AdaBoost) model trained on the optimized feature set. The proposed model achieved an accuracy of 89.3% in the internal validation cohort and demonstrated robust performance in the external validation cohort, with 90.2% sensitivity, 80% specificity, and 88.2% overall accuracy. Comparative analysis with existing radiomics-based studies showed that the proposed model either outperforms or performs on par with the current state-of-the-art methods, particularly in external validation scenarios. These findings highlight the potential of radiomics-driven machine learning approaches in enhancing PCL diagnosis across diverse patient populations.

## 1. Introduction

Pancreatic cystic lesions (PCLs) are being detected with increasing frequency due to advancements in imaging technology and the rising prevalence of an aging population. Studies indicate that PCLs appear in approximately 3% of CT scans [1] and 20–49.1% of abdominal MRIs [2,3,4]. A significant proportion of these lesions are detected incidentally during imaging performed for unrelated conditions, underscoring the importance of early detection and accurate classification to differentiate between benign and potentially malignant lesions [5,6]. Proper characterization of PCLs is essential, as their management varies significantly based on the risk of malignancy. The incidental nature of these findings poses challenges for pancreatic units, requiring comprehensive follow-up strategies, complex therapeutic decision-making, and risk stratification to determine the most appropriate course of action while minimizing unnecessary procedures [7,8,9]. Given these complexities, there is a growing need for advanced diagnostic tools that leverage imaging biomarkers and artificial intelligence to assist in risk assessment and clinical decision-making, ultimately improving patient outcomes.

PCLs, as defined by WHO [10], include non-neoplastic pseudocysts and neoplastic lesions like serous cystic neoplasms (SCN), intraductal papillary mucinous neoplasms (IPMN), and mucinous cystic neoplasms (MCN). While pseudocysts and SCNs are benign, mucinous neoplasms (IPMN and MCN) are clinically significant due to their malignant potential. Studies indicate that up to 70% of IPMNs and 45–65% of MCNs harbor high-grade dysplasia or invasive carcinoma at the time of diagnosis [11,12]. Therefore, differentiating mucinous from non-mucinous PCLs is critical for appropriate clinical management, guiding decisions on surveillance, surgical intervention, or conservative monitoring. Guidelines [7,8,9] recommend surveillance for incidental PCLs without worrisome features, but lesions with concerning characteristics—such as size over 30 mm, solid components, or pancreatic duct dilation—may require further evaluation or intervention [13,14]. CT scans, often the first imaging modality due to accessibility and cost, have lower contrast resolution compared to MRI, making it harder to characterize small or complex cysts. MRI excels in identifying and evaluating small cystic lesions, making it the preferred modality for detailed assessment [15]. Additional tools like endoscopic ultrasound (EUS) or contrast-enhanced EUS (CE-EUS) can aid in further characterization, particularly for detecting malignant features [16,17,18,19].

The integration of Artificial Intelligence (AI) in pancreatic imaging faces challenges due to the organ’s complex anatomy. Although promising, many AI-based approaches remain experimental and require validation [20,21]. Radiomics, which enables high-throughput extraction of quantitative imaging features [22], plays a vital role in supporting decisions across medical applications [23,24], including PCL differentiation [25,26,27,28,29,30,31,32,33,34,35]. This study introduces a software tool that combines radiomic features extracted via PyRadiomics in Python with other radiological parameters such as lesion size, calcifications, and duct contact extracted from the images. Unlike methods focused on classifying specific PCL types [18,36], this tool is designed to address the critical need for accurate mucinous vs. non-mucinous differentiation by integrating diverse imaging markers. Given the malignant potential of mucinous PCLs, improving classification accuracy can aid in early intervention, reducing unnecessary surgeries while ensuring high-risk lesions receive timely treatment. The tool was trained and validated both internally and externally, showing its ability to enhance PCL classification by integrating machine learning with radiomic and radiological data. This innovation aims to improve diagnostic accuracy, streamline decision-making, reduce unnecessary procedures, and identify high-risk lesions earlier, benefiting patient outcomes. Using a dataset from Hospital de Mataró, the study developed and validated an AdaBoost classifier to classify PCLs as mucinous or non-mucinous. Radiologists manually established the ground truth through ROI annotations. Performance metrics such as accuracy, sensitivity, and specificity were used to evaluate the performance of the proposed model on internal and external test sets, with additional analysis comparing radiological, radiomic, and combined features for classification. By explicitly addressing the clinical significance of mucinous PCLs and their malignant potential, this study advances the current state-of-the-art, offering a robust approach for PCL classification and improving diagnostic workflows.

## 2. Related Works

The characterization of pancreatic cystic lesions (PCLs) involves several imaging modalities, such as computed tomography (CT), magnetic resonance imaging (MRI), and endoscopic ultrasound (EUS), each contributing unique advantages in lesion detection and classification [37]. MRI, especially with magnetic resonance cholangiopancreatography (MRCP), is considered the gold standard for PCL assessment due to its superior soft tissue contrast and capability to visualize internal cystic structures, septations, and ductal communication [38]. However, while MRI offers high-resolution anatomical details, its ability to differentiate between mucinous and non-mucinous lesions remains suboptimal, often requiring additional assessment through EUS or contrast-enhanced EUS (CE-EUS) to evaluate cyst wall thickening, mural nodules, and vascular involvement [39,40]. Despite these advancements, morphological overlap between different PCL subtypes remains a significant challenge, leading to inter-observer variability and diagnostic uncertainty [4,41]. Recent studies have explored the potential of radiomics in improving the classification and differentiation of PCLs. Radiomics extracts quantitative features from medical images, capturing morphological, textural, and intensity-based patterns that help characterize PCLs more accurately than conventional imaging alone. To improve diagnostic accuracy, radiomics-based analysis has emerged as a promising tool, allowing for the extraction of quantitative imaging biomarkers from standard radiological scans. Radiomics provides insights into cyst morphology, heterogeneity, and internal structural patterns, thus aiding in lesion classification [42]. Several studies have demonstrated the effectiveness of radiomics in pancreatic lesion characterization, with texture, shape, and intensity-based metrics improving differentiation between various cystic and solid pancreatic tumors [43,44]. For instance, a study in 2022 developed a radiomics model using MDCT scans of surgically resected PCLs, achieving a remarkable AUC of 0.940 for distinguishing between PCL types, outperforming radiologists (AUC: 0.895). The model incorporated 30 features, including both radiological features (age and gender) and radiomic features like wavelet features and texture metrics, with a sensitivity of 97% and specificity of 88% [31]. Another study focused on classifying various types of pancreatic cystic neoplasms, using 1067 features extracted from MDCT scans. Their support vector machine (SVM) classification model achieved an AUC of 0.916, with sensitivities of 83.3% and specificities of 87.6% [29]. Further advancing the application of radiomics, a 2022 study utilized an MMRF ResNet model for classifying serous cystic neoplasms (SCN) and mucinous cystic neoplasms (MCN) in a study of 100 patients, reporting an AUC of 0.93 with excellent sensitivity and specificity [29]. Other studies have demonstrated the potential of radiomics in differentiating PCL subtypes such as SCN, MCN, and IPMN, with models exhibiting strong performance metrics, including high AUC values and sensitivity rates [27,28]. Additionally, a study combined radiological and radiomic features to distinguish between SCN and MCN, achieving an outstanding AUC of 0.994 with high sensitivity (96.8%) and specificity (100%) [26]. These findings highlight the growing role of radiomics and machine learning techniques in enhancing diagnostic accuracy and providing more robust models for the differentiation of PCLs, ultimately leading to improved clinical decision-making. Artificial intelligence (AI) and deep learning approaches are expected to improve automation, robustness, and predictive power, facilitating more precise risk stratification and clinical decision-making [45,46,47].

## 3. Materials and Methods

This section provides a detailed overview of the custom data collection process, pre-processing steps, feature extraction and selection methods, as well as the design and development of the proposed classification model.

### 3.1. Ethics Approval

This observational retrospective multi-center study was conducted in accordance with the Declaration of Helsinki, and approved by the Ethics Committee of Consorci Sanitari del Maresme (protocol code 90/20 and date of approval: 29 April 2021) and Girona (protocol code 2021.190 and date of approval: 25 January 2022). The need to obtain informed consent was waived.

### 3.2. Data Collection and Image Acquisition

A total of 261 contrast-enhanced CT examinations were retrieved from the hospital’s Picture Archiving and Communication System (PACS) in Digital Imaging and Communication on Medicine (DICOM) format and anonymized. The DICOM files were converted into Neuroimaging Informatics Technology Initiative (NIFTI) format using the dicom2nii software version 2.4.11 [34]. The dataset consisted of 171 mucinous and 90 non-mucinous pancreatic cystic lesions (PCLs). Among the mucinous lesions, 98.8% (n = 169) were intraductal papillary mucinous neoplasms (IPMN), and 1.2% (n = 2) were mucinous cystic neoplasms (MCN). The non-mucinous lesions comprised 48.9% (n = 44) pseudocysts and 51.1% (n = 46) serous cystadenomas (SCA). Table 1 summarizes the clinical and demographic characteristics of mucinous and non-mucinous pancreatic cysts. Patients included in this study had histopathology-confirmed pancreatic cystic lesions or incidental PCLs detected during routine imaging, provided they had sufficient follow-up for classification. The dataset was stratified into three cohorts: training (n = 156), validation (n = 28), and external test (n = 77). The training set included 97 mucinous and 59 non-mucinous cases, the validation set had 13 mucinous and 15 non-mucinous cases, and the external test set contained 61 mucinous and 16 non-mucinous cases. The mean age of patients with mucinous cysts was higher across all cohorts (75.99 ± 8.68 years in training, 68.75 ± 13.51 years in validation, and 69.6 ± 8.8 years in the external test cohort) compared to those with non-mucinous cysts (66.1 ± 10.33, 60.73 ± 10.94, and 60.8 ± 7.49 years, respectively). The majority of patients fell within the 61–80 age range (63.4% in training, 50% in validation, and 83.3% in external testing for mucinous cases). Table 2 provides a detailed breakdown of the clinical and demographic characteristics of the training, validation, and external test cohorts. Exclusion criteria included poor-quality scans due to motion artifacts or incomplete pancreas coverage, pseudocysts with a confirmed etiology such as post-pancreatitis, and incidental lesions lacking sufficient follow-up for accurate classification.

### 3.3. Segmentation and Feature Extraction

Three experienced radiologists (N.T., M.D., and Q.G.) with 12, 21, and 7 years of experience, respectively, manually delineated the pancreas and pancreatic cystic lesions (PCLs) on each CT scan using the open-source software 3D Slicer [34]. Segmentation was performed slice by slice to ensure precise annotation of cyst morphology. To assess inter-observer agreement and ensure segmentation consistency, each radiologist independently annotated a subset of images, and discrepancies were resolved through consensus meetings. A subset of segmentations was cross-validated by all three annotators, and cases with disagreement were finalized based on a majority consensus. The study population included patients diagnosed with PCLs through histopathology or long-term imaging follow-up, as well as incidental PCLs identified during routine imaging for other clinical conditions. Control images were selected from patients with no evidence of pancreatic pathology. A comprehensive review of Anatomical Pathology (AP) reports and hospital pancreas committee records was performed to ensure the accurate classification of cystic lesions.

The final dataset consisted of 261 CT examinations with 293 distinct annotations from multiple radiologists. The dataset was compiled following a structured selection protocol, ensuring the inclusion of patients with confirmed PCLs and incidental cystic findings with adequate follow-up. Cases with uncertain diagnoses or insufficient follow-up were excluded. To create a balanced and diverse dataset, the CT scans were categorized into three groups:

1. Training cohort (n = 156): Comprised cases with detailed annotations from three radiologists, including histopathologically confirmed lesions and PCLs verified through imaging follow-up.
2. Internal validation cohort (n = 28): Included newly acquired cases with single-radiologist annotations, used to evaluate model performance on unseen but institutionally similar data.
3. External validation cohort (n = 77): Sourced from an independent institution, this cohort contained single-label annotations and was used to assess model generalizability across different imaging protocols and patient populations.

#### 3.3.1. Preprocessing and Feature Extraction

To ensure consistency in feature extraction and analysis, all CT images and radiologist-provided segmentations underwent a standardized preprocessing pipeline. This process involved two primary steps:

1. Resampling to isotropic space: Voxel dimensions were standardized to mitigate variability caused by differences in slice thickness and pixel spacing, ensuring uniform image representation.
2. Soft tissue normalization: Hounsfield Unit (HU) values were adjusted to a mean of 50 to ensure consistent intensity representation across scans [48].

Following segmentation, the imaging data was processed using a feature extraction pipeline. Feature extraction was categorized into radiological features and radiomic features, both of which played a crucial role in characterizing pancreatic cystic lesions (PCLs) for classification.

Radiological Feature Extraction: Radiological features were manually annotated by three experienced radiologists and included clinically relevant descriptors that are commonly used in pancreatic lesion assessment. These features were selected based on established guidelines and expert consensus to ensure their diagnostic relevance. The extracted radiological features included:

- Lesion volume: The total three-dimensional (3D) volume of the cystic lesion, calculated from the manually segmented region of interest (ROI).
- Lesion position within the pancreas: The anatomical location of the lesion (head, body, or tail of the pancreas).
- Lesion shape: Cyst morphology categorized as either oval, round, or lobulated.
- Lesion size: Maximum cyst diameter measured along its longest axis.
- Calcifications: Presence or absence of calcifications within the cyst or pancreas, which can provide important clues regarding the lesion’s pathology.
- Septations: Presence or absence of internal septations, which may indicate mucinous cystic neoplasms (MCN) or intraductal papillary mucinous neoplasms (IPMN).
- Pancreatic duct communication: Evaluation of whether the cyst has a direct connection to the pancreatic duct, a characteristic commonly associated with IPMNs.

These radiological features formed the ground truth for evaluating the accuracy of the software tool in extracting cyst morphology, structure, and its relationship with pancreatic anatomy.

Radiomic Feature Extraction: Radiomic features were extracted using PyRadiomics, an open-source radiomics toolkit that enables high-throughput computation of quantitative imaging features. These features provide insights into the texture, intensity, and spatial distribution of pixel values within the segmented lesion. The extracted radiomic features were derived from the following matrices:

- Gray Level Co-occurrence Matrix (GLCM): Measures the spatial relationship between pixel intensities to capture texture homogeneity, contrast, and correlation.
- Gray Level Run Length Matrix (GLRLM): Evaluates the length of consecutive pixels with the same gray level, providing information about coarseness and granularity.
- Gray Level Size Zone Matrix (GLSZM): Quantifies the size distribution of regions with similar gray levels, highlighting patterns of heterogeneity and uniformity within the lesion.

**Figure 1:**
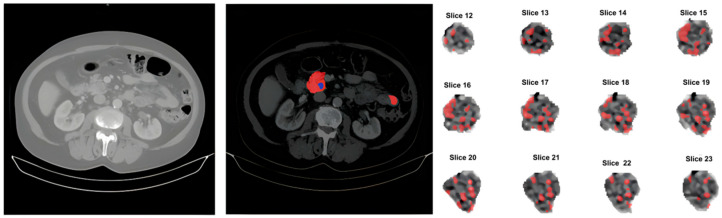
Processing pipeline. Illustration of the original study (**left** image), output of the soft-tissue normalization and manual segmentation of the pancreas and lesions, red and blue respectively (middle image) and image feature extraction of the segmented lesion (**right** image).

These radiomic features were extracted to complement radiological descriptors, allowing for a more comprehensive quantitative analysis of lesion characteristics. Figure 1 illustrates the preprocessing pipeline.

#### 3.3.2. Feature Selection and Dimensionality Reduction

Given the high dimensionality of radiomic features, a systematic feature selection process was employed to retain only the most informative and discriminative predictors for classification. A Least Absolute Shrinkage and Selection Operator (LASSO) regression model with L1 regularization was applied to eliminate non-contributory features by assigning zero coefficients to those with minimal relevance. This approach effectively reduced feature redundancy, minimized overfitting, and improved model generalization. To further refine the selection process, a correlation threshold of 0.8 was applied to exclude highly collinear features, ensuring that only independent and clinically meaningful predictors were retained. Selecting a LASSO coefficient threshold of 0.8 maximized accuracy in the internal validation cohort while preserving 60% of the total feature set, striking a balance between dimensionality reduction and predictive performance. Table 3 presents the radiological and radiomic features systematically organized into eight categories, one for radiology and seven for radiomics. Each category includes a comprehensive description of its significance, with the fourth column highlighting the specific selected features used for classification when applying the 0.8 threshold. This structured feature selection process allowed for the retention of the most relevant attributes, ultimately contributing to a robust model that is both accurate and interpretable.

### 3.4. Machine Learning Model Development

To classify pancreatic cystic lesions (PCLs) as mucinous or non-mucinous, we developed a machine learning model based on Adaptive Boosting (AdaBoost). AdaBoost was chosen over alternative classifiers, such as Support Vector Machines (SVM) and Random Forest, due to its ability to handle high-dimensional datasets, focus on misclassified instances through iterative learning, and minimize overfitting by combining multiple weak classifiers into a strong predictive model. Additionally, AdaBoost has demonstrated superior performance in imbalanced datasets, ensuring robust learning even when feature distributions vary.

The model was trained on a dataset containing the most relevant radiological and radiomic features, as identified through Least Absolute Shrinkage and Selection Operator (LASSO) regression as discussed in the previous section. To optimize performance, hyperparameter tuning was conducted, resulting in a learning rate of 0.3, a maximum tree depth of 1, and 50 estimators. The model was implemented using the scikit-learn library in Python. The model was trained using a dataset that included the refined subset of the most relevant features identified through the feature selection process. To assess its performance in detecting pancreatic mucinous cysts, the model was validated using an independent validation cohort. Model performance was evaluated by comparing the predictions against the ground truth annotations. Standard classification metrics, including accuracy, sensitivity, specificity, precision, F1-score, and the Area Under the Curve (AUC) of the Receiver Operating Characteristic (ROC) curve, were calculated to quantify the model’s effectiveness.

**Figure 2:**
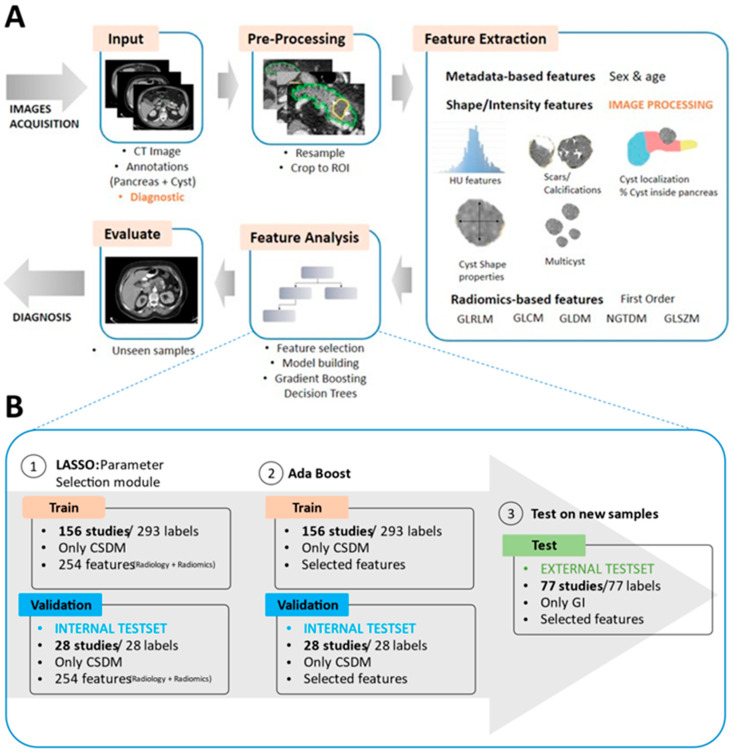
Methodology for pancreatic cyst classification algorithm based on radiological and radiomics features. (**A**) Comprehensive workflow of the methodology employed for developing a classification algorithm based on radiological and radiomics features. The process begins by defining inputs, which include CT images with corresponding pancreas and cyst segmentations, along with diagnoses indicating whether the lesion is mucinous (1) or non-mucinous (0). Pre-processing is applied to these images to reduce unnecessary computational loads. Image processing techniques are then utilized on lesion segmentations to extract the initial subset of features. Subsequently, a feature analysis procedure is executed to identify the most critical features, forming the foundation for constructing the final diagnosis model. The final diagnosis model is designed for subsequent generalization to unseen datasets. (**B**) Organization of the dataset during the feature selection step, encompassing three sub-steps: LASSO selection of the most important features, construction of the final model using the selected features, and evaluation on the external testing set.

In addition to radiomic feature extraction, a comprehensive radiological analysis was performed by three experienced radiologists who evaluated key imaging characteristics through a consensus review. The radiological assessment focused on important features such as lesion shape, size, multiplicity, anatomical location (head, body, or tail of the pancreas), presence of calcifications, septations, mural nodules, and pancreatic duct communication (contact or no contact) [49,50,51]. These radiological features were integrated into the classification framework to enhance model interpretability and improve diagnostic accuracy. By combining both radiological and radiomic feature analysis, this approach ensures a robust and precise diagnostic framework for classifying pancreatic cystic lesions. It not only improves the classification performance but also supports better decision-making in a clinical setting. The overall workflow of the proposed machine learning architecture is illustrated in Figure 2.

### 3.5. Statistical Analysis

The Kolmogorov–Smirnov test was used to assess normality. Categorical variables are presented as total number and percentages and continuous variables are shown as median values and interquartile ranges (IQR) for non-normally distributed data, or by means and standard deviations (SD) for normally distributed data. The chi-square (χ^2^) test was used to analyze categorical variable group differences, while the *t*-test and Mann–Whitney U test were utilized for continuous variables based on their distribution. The results were considered as statistically significant when the calculated *p*-value was less than 0.05. Statistical analyses were conducted with IBM SPSS Statistics (version 29.0.0.0).

To evaluate the performance of the machine learning model in differentiating mucinous from non-mucinous lesions, we employed several metrics. Accuracy, calculated as $\frac{TP+TN}{FP+FN+TP+TN}$, where TP represents true positives, TN true negatives, FP false positives, and FN false negatives, was used to assess the overall accuracy of the model. Sensitivity or recall, determined by $\frac{TP}{FN+TP}$, determined the model’s ability to correctly identify positive cases. Specificity, defined as $\frac{TN}{FP+TN}$, evaluated the model’s capacity to correctly identify negative cases. Precision, calculated as $\frac{TP}{TP+FP}$, assessed the proportion of true positive results among all positive results. The F1-Score, given by $2\;\cdot \frac{PrPr\;e\;cision\;\cdot \;Recall}{PrPr\;e\;cision\;+\;Recall}$, calculates the harmonic mean between precision and recall. Lastly, the Area Under the Curve (AUC) of the Receiver Operating Characteristic (ROC) curve was used to evaluate the model’s ability to differentiate mucinous and non-mucinous lesions across different threshold settings.

## 4. Results

### 4.1. Study Population

We included a total of 261 CT scans from 106 patients. Among these, 156 scans were part of the training set, 28 comprised the internal validation test, and 77 were in the external test set. In our study, we identified four major subtypes, classifying them into non-mucinous lesions (serous cystic neoplasm, pseudocyst) and mucinous lesions (mucinous cystic neoplasm, and intraductal papillary mucinous neoplasm (IPMN)).

Among the total CT scans, 171 were classified as mucinous and 90 as non-mucinous. The prevalence of mucinous lesions was found to increase with age, with mucinous cases showing a significantly higher median age (*p* < 0.001). When stratified into training, validation, and external test cohorts, the demographic distribution remained largely consistent, with age, sex, and the mean number of follow-up CTs per patient being comparable across these groups (*p* = 0.083, *p* = 0.087, and *p* = 0.091, respectively) as detailed in Table 2. Specifically, the trainset identified 97 mucinous and 59 non-mucinous lesions on CT from 42 and 23 patients. The internal test set included 13 mucinous and 15 non-mucinous PCL from 10 and 7 patients, while the external test set comprised 61 mucinous and 16 non-mucinous PCL from 19 and 5 patients, respectively (Table 2).

### 4.2. Analysis of Standard Radiological Characteristics

**Figure 3:**
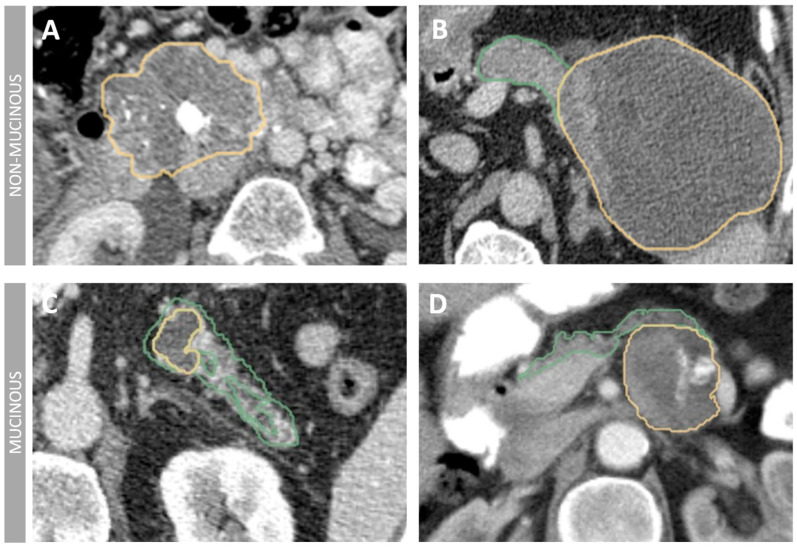
Imaging features of PCLs detected by CT. (**A**) Patient with a 76 mm serous cystic neoplasm (SCN) in the head of the pancreas. Enhancing septations, peripheral calcifications and central scar with calcification. (**B**) Well-defined 122 mm pseudocyst in the tail of the pancreas with denser areas, which may indicate the presence of blood or hemorrhage within the cyst. (**C**) Intraductal Papillary Mucinous Neoplasm (IPMN) measuring 33 mm in the pancreatic body, displaying communication with the main pancreatic duct, which is dilated (7 mm). (**D**) Illustration of a mucinous cystic neoplasm located in the body-tail of the pancreas. The lesion measures 49 mm and exhibits septae along with thick walls, indicative of its characteristic features. Pancreatic cystic lesions are depicted in yellow, while the pancreas is highlighted in green.

**Figure 4:**
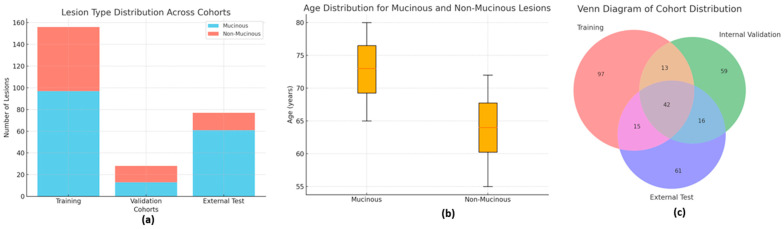
Distribution and Characteristics of Mucinous and Non-Mucinous Lesions Across Cohorts. (**a**) Proportions of mucinous and non-mucinous lesions within the training, internal validation, and external test sets, demonstrating consistency across datasets. (**b**) Age distribution of patients with mucinous and non-mucinous lesions, showing a significantly higher median age for the mucinous group (*p* < 0.001). (**c**) Venn diagram illustrating the overlap of patient samples across the three cohorts, highlighting dataset composition and shared cases.

Our study classified PCL into non-mucinous (SCN, pseudocyst) and mucinous (MCN, IPMN) subtypes based on radiological characteristics (Figure 3). Table 4 summarizes the measured standard radiological characteristics. Mucinous cysts exhibited a higher prevalence of multiple cysts, and smaller size, with cysts typically measuring less than 30 mm (*p* < 0.001). In contrast, non-mucinous cysts were characterized by a greater presence of septa, scars, and cystic calcifications and pancreatic (*p* < 0.05). Mucinous cysts were more likely to exhibit communication or contact with the pancreatic duct (*p* < 0.001). Subsequently, we integrated these radiological features into the radiological model. Figure 4a visualizes the distribution of mucinous and non-mucinous lesions across the three cohorts. It shows the relative proportions of mucinous versus non-mucinous lesions within the training, internal validation, and external test sets. This representation clearly illustrates the consistency of lesion types across the datasets. Figure 4b depicts the age distribution for mucinous and non-mucinous lesions. The boxplot reveals that mucinous lesions tend to affect older patients, with a significantly higher median age for the mucinous group compared to the non-mucinous group (*p* < 0.001). This finding underscores the demographic differences in the distribution of these lesions. Figure 4c presents a Venn diagram illustrating the overlap of patient samples across the three cohorts. It provides a clear view of the number of unique and shared patients between the training, internal validation, and external test sets, highlighting the distribution and composition of the datasets.

### 4.3. Feature Extraction in Radiomics Analysis

The study involved 184 CT examinations from Hospital de Mataró, divided into a training cohort (156 CT) and an independent validation cohort (28 CT). An external testing cohort from Hospital Universitari Dr. Josep Trueta comprised 77 CT. In order to provide more trustworthiness to the results presented by the method, we propose an external blind validation where the testset is composed by CT scans from a different hospital and different manufacturers than the ones used for the training. To achieve this, in this paper we present a training set formed by 156 CT examinations gender balanced and heterogeneous composed by a 60% of mucinous lesions (IPMNs and MCNs) and a 40% of non-mucinous lesions (Pseudocysts and SCAs). All these studies were labeled and their features (presence of calcifications, size, volume, etc.) were manually analyzed by the experienced radiologists involved in this study.

As mentioned above, the test set is formed by two groups of CT examinations: 28 CT extracted from the same institution of the studies included in the training set but belonging to patients that are not included in the training, to avoid potential bias in the results. Moreover, the testing set is completed with 77 CT examinations from a different Institution (35% of non-mucinous lesions and 65% belong to mucinous lesions). For each CT examination, the pancreas and the margin of each PCL were manually outlined (region of interest; ROI). This precise manual delineation ensured accurate analysis, eliminating potential artifacts and ensuring robust data for the study. A total of 379 studies with manually labeled ROIs served as the dataset for training and testing the algorithm.

Following ROI segmentation in radiological images, the next step involved feature extraction. The aim was to identify pertinent information without prior clinical hypotheses, characterizing both normal and abnormal regions. Extracted features were categorized into two groups: radiomics and radiological features. Radiomics features included agnostic or non-semantic elements, enabling the discovery of previously unseen image patterns for classification or prediction. These features were derived mathematically, as above-mentioned, using Python package PyRadiomics [52]. Initially, key statistical metrics such as minimum, maximum, mean, and standard deviation were computed for the Hounsfield Unit (HU) values. Additionally, this first set of features was supplemented with other parameters derived from commonly known radiomics matrices. A total amount of 214 features were obtained for the pancreas and cyst regions (Figure 2).

After obtaining the radiomics features, a subsequent set of features was extracted through image processing and segmentation methods. This second set encompassed various aspects, including the precise localization of the cyst in relation to the pancreas. PCLs located in different regions of the pancreas, such as the head, body, or tail, were differentiated. Additionally, segmentation of scars and calcifications was performed. Moreover, the size and shape of the cysts were analyzed to gain a comprehensive understanding of their morphology. The relative location of the cysts concerning the centerline of the pancreas, approximating the position relative to the pancreatic duct, was also taken into consideration. Incorporating metadata features, such as age and sex, was also considered essential, as these demographic parameters can provide valuable context for the analysis.

**Figure 5:**
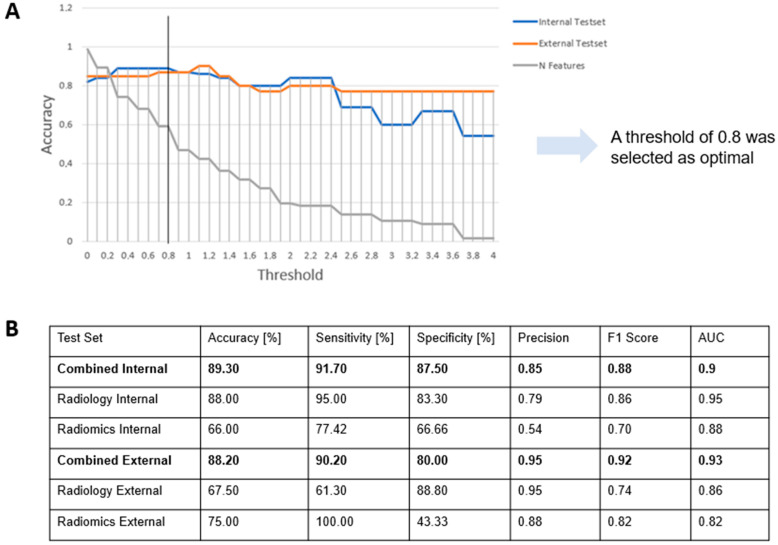
Internal and external validation results. (**A**) Internal and external validation cohorts’ accuracy evolution with respect to the threshold to filter parameters depending on their LASSO coefficients. A threshold of 0.8 is selected as optimal as maximizing internal validation cohort accuracy, keeping a 60% of the total number of features. (**B**) Comparative analysis of the model performance metrics on the test set, with results categorized into internal and external datasets. The evaluation encompasses key performance indicators, including Accuracy, Sensitivity, Specificity, Precision, F1 Score, and Area Under the Curve (AUC).

After acquiring both the radiomics and radiological features from all the CT examinations utilized in this study, a parameter filtering process was carried out to extract the most statistically significant features for the final classification algorithm. To accomplish this, a LASSO regressor was employed as the feature selector which assigns a coefficient to each of the input features. Different thresholds were evaluated to filter out less important features. A threshold of 0.8 was selected as giving the best results in our internal validation dataset, as shown in Figure 5.

### 4.4. Algorithm Performance Evaluation

The algorithm’s ability to distinguish mucinous and non-mucinous PCL was assessed using a dataset from Hospital de Mataró, which included a training cohort (156 CT) and an internal testing cohort (28 CT). An additional external validation cohort from Hospital Universitari Dr. Josep Trueta comprised 77 CT examinations. The initial test set (27 CT) served for parameter optimization and wasn’t meant to assess the model’s generalizability, with its relatively smaller size not significantly impacting result validity.

The combined algorithm, integrating both radiological and radiomics features, demonstrated a sensitivity of 91.7% and specificity of 87.5% in correctly identifying mucinous lesions in the internal validation cohort, giving an accuracy of 89.3%. Furthermore, when tested on the external independent validation cohort including 77 external CT examinations, the algorithm exhibited a sensitivity of 90.2% and a specificity of 80%, resulting in an overall accuracy of 88.2%. This represents a significant improvement when compared to the 75.0% of accuracy achieved when relying solely on radiomics features (100% sensitivity and 43.33% specificity), and the 67.5% of accuracy when relying on radiological features (61.3% sensitivity and 88.8% specificity), indicating that the integration of both radiological and radiomics features enhances the model’s generalization and robustness. Additionally, for the internal test set, the algorithm showed a precision, F1 score, and AUC of 0.85, 0.88, and 0.90, respectively. For the external test set, these metrics were 0.95, 0.92, and 0.93, underscoring the algorithm’s high performance and reliability across different datasets. The achieved accuracy rates in both internal and external validation cohorts highlight the robustness and generalizability of our algorithm. The integration of radiological and radiomics features significantly improved diagnostic performance compared to using either feature set alone. For instance, relying solely on radiomics features yielded an accuracy of 75.0%, while using only radiological features resulted in an accuracy of 67.5%. This underscores the synergistic effect of combining both feature types, which enhances the model’s ability to differentiate between mucinous and non-mucinous lesions across diverse datasets.

Moreover, individual accuracies for specific radiological parameters used in model training were calculated. Our radiology feature extraction algorithm, based on image processing, achieved an 88.78% accuracy for scars detection (66.66% sensitivity and 89.40% specificity), an 82.50% accuracy for cyst calcifications (84.00% sensitivity and 82.32% specificity), and an 82.05% accuracy for cyst location within the pancreas (head, body, or tail). Furthermore, the accuracy in discriminating multicystic lesions reached 71.30%. These results provide further insights into the algorithm’s capability to effectively utilize and differentiate specific radiological features, contributing to its overall success in lesion identification and classification.

### 4.5. Exploring Pancreatic Cystic Lesions Analysis Through Diverse Radiomic Studies: A Comparative Perspective

To reinforce our findings and perform a comparative analysis, we conducted a thorough literature review by searching PubMed using the keywords “Radiomics” and “pancreatic cystic lesions. This search identified 10 studies utilizing radiomics or ML for PCL differentiation (Table 5). Recent research consistently demonstrates the superiority of radiomics and ML over traditional radiological methods in PCL classification, showcasing their potential to enhance diagnostic accuracy across diverse patient cohorts. In Wei et al. [23], a radiomics-based diagnostic scheme outperformed clinician assessments, achieving an AUC of 0.837, sensitivity of 0.667, and specificity of 0.818 for PCN. Xie et al. [26] presented robust diagnostic capabilities with radiological and radiomics models. The radiological model achieved an AUC of 0.775, sensitivity of 74.2%, and specificity of 80.8%, while the radiomics model excelled with an AUC of 0.989, sensitivity of 93.6%, and specificity of 96.2%. Yang et al. [22] effectively differentiated PCL subtypes using CT-derived textural parameters, achieving AUCs of 0.77 in the training group and 0.66 to 0.75 in the validation group. Liang et al. [30] developed a classification model for serous cystic neoplasms (SCNs) and mucinous cystic neoplasms (MCNs), achieving an AUC of 0.93, sensitivity of 91.63%, and specificity of 93.80%. In 2017, Dmitriev et al. [33] introduced an algorithm with an 83.6% accuracy in categorizing common PCL types. Shen et al. [25] effectively employed ML to classify PCL subtypes, demonstrating notable success with a precision of 90% in MCNs and 72% in SCNs, highlighting a higher accuracy in identifying mucinous lesions over serous cystic neoplasms. Evaluating 488 radiomic features, Chu et al. [31] surpassed the performance of radiologists in distinguishing between different PCLs, and Yang et al. [29] (2022) showcased the effectiveness of the MMRF-ResNet model in accurately classifying pancreatic SCNs and MCNs. Interestingly, Chen et al. [28] developed a nomogram achieving high accuracy in differentiating between SCNs and mucin-producing PCN. Additionally, Xu & He [27] integrated radiomics with preoperative variables, achieving accurate classification of SCNs, mucinous cystadenomas, and IPMNs.

**Figure 6:**
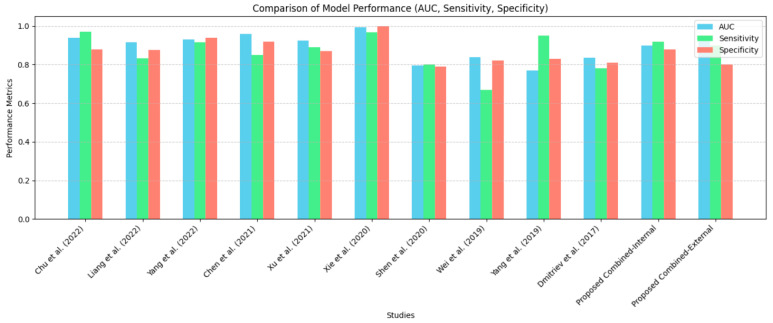
Comparative performance of radiomics models for PCL classification. Studies integrating radiomics with radiological features including the proposed model demonstrate balanced sensitivity and specificity [22,26,27,28,29,30,31,32,33,34].

**Figure 7:**
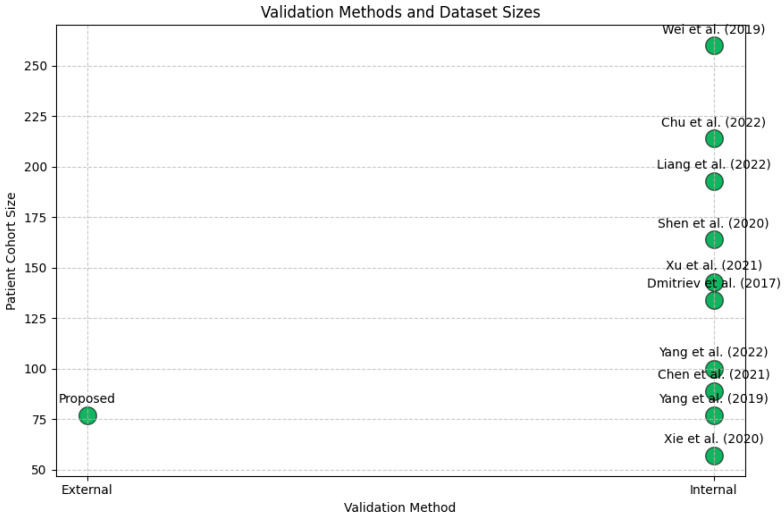
Validation Methods in Radiomics Studies: Internal vs. External Cohorts [22,26,27,28,29,30,31,32,33,34].

Figure 6 compares the performance of the proposed model against state-of-the-art methods in terms of AUC score, sensitivity, and specificity. The results indicate that the proposed model demonstrates competitive performance, achieving similar or better metrics compared to existing approaches. Notably, the proposed model with external validation maintains strong AUC, sensitivity, and specificity, reinforcing its reliability. To further evaluate generalizability, Figure 7 presents a bubble plot illustrating validation methods and dataset sizes in radiomics studies. The x-axis differentiates between internal and external validation, while the y-axis represents patient cohort size, with bubble size corresponding to the cohort size of each study. The plot highlights that most studies predominantly rely on internal validation, with only one study employing external validation, emphasizing the limited use of external cohorts in radiomics research. Even larger datasets, such as Wei et al. (2019) [26] with n = 260, continue to use internal validation. In contrast, the proposed study is among the few that incorporate external validation, underscoring the need for broader validation practices to enhance the generalizability and clinical applicability of radiomics models.at most studies rely on internal validation, with only one study employing external validation, emphasizing the limited use of external cohorts in radiomics research. Notably, even larger datasets, such as Wei et al. (2019) [26] with n = 260, continue to use internal validation. In contrast, the proposed study is among the few utilizing external validation, underscoring the need for broader validation practices to enhance the generalizability of radiomics models.

## 5. Discussion

Classifying PCLs is complex due to varying malignancy risks and limitations in traditional imaging, which can lead to misdiagnosis or overtreatment. While MRI is preferred for its superior soft-tissue resolution and detailed cyst chracterization, many PCLs are initially detected on CT scans, which are commonly used as the first diagnostic tool. However, CT scans demonstrate variable accuracy (40–81%) in differentiating benign and malignant PCLs [14,53,54,55,56,57,58,59,60], and their performance in identifying specific types, such as serous cystic neoplasms [58], remains limited. This diagnostic variability underscores the need for more precise and reliable tools.

Our study addresses this gap by evaluating a radiomics-based tool designed to classify PCLs as mucinous or non-mucinous using CT scan data. Through a retrospective analysis of 184 CT examinations from Hospital de Mataró, coupled with external validation cohorts from Hospital Universitari Dr. Josep Trueta, the algorithm demonstrated strong performance metrics. In the internal validation set, the algorithm achieved a sensitivity of 91.7%, specificity of 87.5%, and accuracy of 89.3%. External validation confirmed these findings, with sensitivity, specificity, and accuracy rates of 90.2%, 80%, and 88.2%, respectively. Additionally, performance metrics such as precision (0.95), F1 score (0.92), and AUC (0.93) in the external cohort further highlighted its robustness and reliability.

The inclusion of external validation cohorts is a key strength, enhancing the tool’s generalizability beyond the internal dataset as shown in Figure 7. The limited cohort diversity may restrict the model’s broad applicability across different patient populations, imaging protocols, and clinical settings. However, the study is limited by its relatively small sample size and retrospective design. The relatively limited diversity of the cohorts also restricts the broad applicability of the findings. Future studies should focus on larger, multicenter datasets and prospective validations to strengthen the tool’s clinical utility and ensure its applicability across diverse patient populations. Further more bias detection techniques, such as SHAP and GradCAM, can help identify unintended correlations in the data. Additionally, advanced learning methods, including domain adaptation, transfer learning, and federated learning, can improve model robustness across different imaging settings. Finally, prospective clinical trials are essential to validate the model’s real-world performance beyond retrospective analysis.

The clinical impact of this radiomics-based tool could be profound. By improving the accuracy of PCL classification, it can help clinicians make more informed decisions, leading to more tailored and appropriate treatments for patients. For instance, distinguishing between mucinous and non-mucinous lesions with greater precision can aid in better risk stratification, potentially guiding decisions regarding the need for surveillance versus intervention. In the case of mucinous lesions, which are more likely to become malignant, early and accurate identification could lead to timely surgical interventions, reducing the risk of progression to pancreatic cancer. Conversely, non-mucinous lesions, which are typically benign, may avoid unnecessary interventions, thus reducing the risk of overtreatment and patient morbidity. Furthermore, integrating radiomics and machine learning into PCL diagnosis represents a significant advancement over traditional methods, which often lack the precision required for accurate risk assessment. This approach combines radiomic feature extraction with advanced classification algorithms, enabling the identification of meaningful imaging features such as lesion volume, position within the pancreas, and texture-based radiomic parameters. These features improve the accuracy of PCL classification and, in turn, can lead to better clinical outcomes by enabling clinicians to make decisions based on more reliable, data-driven insights. By leveraging the strengths of radiomics and machine learning, this tool has the potential to enhance the efficiency and accuracy of PCL diagnosis, ultimately improving patient care and reducing healthcare costs. This further emphasizes the importance of advancing research in this area, focusing on larger-scale studies and prospective clinical trials to validate the tool’s impact in real-world clinical settings.

## 6. Conclusions

In summary, this research provides compelling evidence for the application value of radiomics in differentiating pancreatic cystic lesions. Our study demonstrates that an innovative ML-based tool can accurately categorize PCLs, with the algorithm showing impressive sensitivity, specificity, and overall accuracy. The inclusion of an external validation cohort is a significant strength, ensuring the algorithm’s robustness and generalizability beyond internal datasets. However, we recognize the limitations of a small sample size and advocate for future research to expand external validation cohorts with diverse hospital data to improve generalizability and statistical power in real-world clinical scenarios. We anticipate that future studies developing more robust predictive models will significantly enhance the role of artificial intelligence in aiding clinicians with disease diagnosis and treatment. Extensive prospective studies are essential to further validate the diagnostic accuracy and clinical utility of radiomics.

## Figures and Tables

**Table 1:** Clinical and demographic characteristics of mucinous and non-mucinous pancreatic cysts. Categorical variables are shown as total numbers and percentages (%), non-parametric continuous variables as medians (interquartile ranges) and means ± SD for normally distributed variables. Pancreatic cyst lesions were categorized into mucinous or non-mucinous. Statistical significance was assessed using χ2 for categorical variables and *t*-tests or Mann–Whitney U tests for continuous variables. IPMN, intraductal papillary mucinous neoplasms; MCN, mucinous cystic neoplasms; SCA, serous cystadenoma.

	Mucinous (n = 171)	Non-Mucinous (n = 90)	
IPMN, n (%)	169 (98.8%)	-	
MCN, n (%)	2 (1.2%)	-	
Pseudocyst, n (%)	-	44 (48.9%)	
SCA, n (%)	-	46 (51.1%)	
			*p* value
Total of patients	71	35	
Age, years	75 (69–80)	65 (58–70)	<0.001
0–18, n (%)	0	0	
19–40, n (%)	0	2 (2.3%)	
41–60, n (%)	9 (6.3%)	26 (29.9%)	
61–80, n (%)	100 (69.4%)	55 (63.2%)	
81–100, n (%)	35 (24.3%)	4 (4.6%)	
Women, n (%)	59 (39.9%)	32 (37.2%)	0.688
Mean n of follow-up CTs/patient	2.47 ± 2.03	2.57 ± 1.61	0.799

**Table 2:** Clinical and demographic characteristics of mucinous and non-mucinous pancreatic cysts in training, validation, and external test cohort. Categorical variables are shown as total numbers and percentages (%), non-parametric continuous variables as medians (interquartile ranges) and means ± SD for normally distributed variables. Pancreatic cysts were categorized into mucinous or non-mucinous categorized into the training, validation, and external test cohorts. IPMN, intraductal papillary mucinous neoplasms; MCN, mucinous cystic neoplasms; SCA, serous cystadenoma.

	Train (n = 156)		Validation (n = 28)		External Test (n = 77)	
	Mucinous (n = 97)	Non-Mucinous (n = 59)	Mucinous (n = 13)	Non-Mucinous (n = 15)	Mucinous (n = 61)	Non-Mucinous (n = 16)
IPMN	96 (99%)		12 (92.3%)		61 (100%)	
NQM	1 (1%)		1 (7.7%)		0	
Pseudocyst		28 (47.5%)		8 (53.3%)		8 (50%)
SCA		31 (52.5%)		7 (46.7%)		8 (50%)
Age	75.99 ± 8.68	66.1 ± 10.33	68.75 ± 13.51	60.73 ± 10.94	69.6 ± 8.8	60.8 ± 7.49
0–18	0	0	0	0	0	0
19–40	0	1 (1.7%)	0	1 (6.7%)	0	0
41–60	5 (5.4%)	16 (27.6%)	3 (25%)	4 (26.7%)	4 (9.5%)	6 (42.9%)
61–80	59 (63.4%)	37 (63.8%)	6 (50%)	10 (66.7%)	35 (83.3%)	8 (57.1%)
81–100	29 (31.2%)	4 (6.9%)	3 (25%)	0	3 (7.1%)	0
Women	43 (45.7%)	22 (37.9%)	8 (61.5%)	4 (28.6%)	9 (20.5%)	6 (42.9%)
Total of patients	42	23	10	7	19	5
Mean n of follow-up CTs/patient	2.31 ± 1.6	2.57 ± 1.65	1.3 ± 0.48	2.14 ± 1.07	3.21 ± 2.89	3.2 ± 2.17

**Table 3:** Radiological and radiomic features. Description and selection. Selected features for the classification step. Systematically organized into 8 categories, comprising 1 for radiology and 7 for radiomics. Each category is accompanied by a comprehensive description of its significance, with the fourth column detailing the specific selected features pertinent to each classification when applying the 0.8 threshold.

	Class	Description	Selected Features
Radiology	Radiological	Guideline-based features.	Age, Sex, Cyst minor axis, Scars, HU Scars, Pancreas and cyst calcifications, Air balls, % of cyst in Head, Distance of cyst with respect to central pancreatic line, #Pixels cyst inside pancreas, % of cyst inside pancreas, %Solid, #Pixels pancreas, Big Lobes, min. HU Cyst, mean HU Cyst, diff. HU Cyst-Pancreas.
Radiomics	Shape-based	Geometric descriptors characterizing lesion shapes.	Mesh Volume, Elongation, Flatness, Surface Area, Voxel Volume, Sphericity, Major Axis Length, Least Axis Length, Maximum 2D Diameter Row.
	First-Order	Immediate statistical metrics, like average and deviation, offering insights into pixel intensity.	Root mean squared, Kurtosis, Robust Mean Absolute Deviation, Range, Maximum, 10 Percentile, Interquartile Range.
	Gray Level Co-occurrence Matrix (GLCM)	Analyzes the spatial relationship of gray levels in the image.	Cluster Prominence, Contrast, Idn, Imc1, Imc2, MCC.
	Gray Level Run Length Matrix (GLRLM)	Measures the length of sequences of pixels with the same gray level.	RunEntropy, Short Run High Gray Level Emphasis, Short Run Low Gray Level Emphasis, High Gray Level Run Emphasis, Short Run Emphasis, Run Length Non Uniformity Normalized.
	Gray Level Size Zone Matrix (GLSZM)	Examines the size of regions with similar gray levels, highlighting size distribution.	Zone Entropy, Low Gray Level Zone Emphasis, Large Area Low Gray Level Emphasis, Gray Level Non Uniformity Norm., Small Area Emphasis.
	Neighbouring Gray Tone Difference Matrix (NGTDM)	Quantifies the difference between a gray-value and the average gray-value of its neighbours, offering insights into local changes.	Contrast
	Gray Level Dependence Matrix (GLDM)	Characterizes the dependence between gray levels of neighboring pixels, highlighting spatial dependence patterns.	High Gray Level Emphasis, Dependence Variance, Dependence Entropy, Small Dependence Emphasis.

**Table 4:** Radiological characteristics of mucinous and non-mucinous pancreatic cysts. Categorical variables are shown as total numbers and percentages (%), non-parametric continuous variables as medians (interquartile ranges) and means ± SD for normally distributed variables. Pancreatic cysts were categorized into mucinous or non-mucinous. Statistical significance was assessed using χ2 for categorical variables and *t*-tests or Mann–Whitney U tests for continuous variables. IPMN, intraductal papillary mucinous neoplasms; PCL, pancreatic cystic lesions; SCA, serous cystadenoma. * Undetermined/multiple refers to some cyst locations that could not be defined or that multiple cysts were found in different locations in the same scan.

Features	Mucinous	Non-Mucinous	
Location			<0.001
Head	42 (24.6%)	44 (48.9%)	
Body	33 (19.3%)	12 (13.3%)	
Tail	23 (13.5%)	20 (22.2%)	
Undetermined/multiple *	73 (42.7%)	14 (15.6%)	
Cysts			<0.001
Solitary	98 (57.3%)	76 (84.4%)	
Multiple	73 (42.7%)	14 (1.2%)	
Largest cyst size (mm)	17 (13–24)	38 (21–73)	<0.001
Bigger cysts			<0.001
<3 cm	120 (82.2%)	35 (39.3%)	
≥3 cm	26 (17.8%)	54 (60.7%)	
Lobulations	16 (9.4%)	13 (14.4%)	0.214
Septa	35 (20.6%)	45 (50%)	<0.001
Scars	0	6 (6.7%)	0.001
Cystic calcifications	1 (0.6%)	13 (25.6%)	<0.001
Pancreatic calcifications	13 (7.6%)	14 (15.6%)	0.047
Communication/contact with the pancreatic duct	113 (69.3%)	26 (28.9%)	<0.001
Pancreatic duct dilatation	36 (21.2%)	19 (21.3%)	0.974

**Table 5:** Comprehensive assessment of articles analyzed using radiomics for PCL classification and differentiation. LASSO (least absolute shrinkage selection operator); SVM Support Vector Machine, NGTDM Neighborhood gray tone difference matrix; GLCM Gray-level; co-occurrence matrix, NGLDM Neighborhood gray-level different matrix, GLRLM Gray level run length matrix; GLZLM Gray level zone length matrix, LBP local binary pattern PCL: Pancreatic cystic lesion. RF: Random Forest. CNN: Convolutional neural network. ANN: Artificial neural networks. MMRF: Multichannel-Multiclassifier-Random Forest.

Author	Primary Outcome	Study Type	Years	Patients (N)	Imaging Modality	Radiomic Features (N)	Features Selected	Model	Validation	Performance
Chu, USA, 2022 [30]	Discriminate PCL type radiomics vs. radiologist.	· Retrospective · Surgically resected PCL	2003–2016	214 (64 IPMN, 33, 60, 24, 33)	MDCT	488	30 features: · 2 Radiological features: age and gender. · 28 Radiomics features: 12 wavelet features, 7 from the Laplacian of Gaussian (LoG) filtered volume and 9 other textural or morphological image features.	Random Forest	Internal	AUC: 0.940 vs. 0.895 (radiologist) Sn: 97% Sp: 88%
Liang, China, 2022 [31]	Classification prediction of pancreatic cystic neoplasms	· Retrospective	2012–2020	193 (99 SCA, 55 MCA, 39 IPMN)	MDCT	1067	83 features: · 10 Radiological features: tumor position, maximum diameter, unilocular/ multilocular cysts, lobulation, polycystic features (≥2, ≥6), nodular soft tissue, cystic wall calcification, communication with the pancreatic duct, pancreatic duct dilatation, and peripancreatic lymph node enlargement. · 73 Radiomics features: 2 global histogram features, 2 texture features, 12 LBP features and 57 wavelet features.	SVM classification algorithm	Internal	AUC: 0.916 Sn: 83.3% Sp: 87.6%
Yang, China, 2022 [32]	Classification model of SCN and MCN	· Retrospective · Surgically resected PCL	2010–2016	100 (63 SCN, 47 MCN)	MDCT	N/A	N/A	MMRF ResNet	Internal	AUC: 0.93 Sn: 91.63%, Sp: 93.80%,
Chen, China, 2021 [33]	Differential diagnosis of SCNs from mucin producing PCNs.	· Retrospective	2013–2019	89 (31 SCNs, 30 IPMNs, and 28 MCNs)	MDCT	710	16 features: · 8 Radiological features: gender, age, location of tumors, contour of tumors, tumor margin, calcification, multilocular, main pancreatic duct. · 8 Radiomics features: among 9 morphological, 42 histogram, 288 GLCM, 360 GLRLM and 11 GLSZM features, a small subset of 8 features were selected.	LASSO	Internal	AUC: 0.960
Xu, China, 2021 [34]	Preoperative Differentiation: SCN, MCN And IPMN	· Retrospective · Surgically resected PCL	2016–2020	143	MDCT	1218	836 features: · 3 Radiological features · Among 1218 radiomic features including 16 shape descriptors, 19 first-order statistics, 75 texture features and 688 wavelet descompositions,833 of them were selected.	LASSO	Internal	AUC: 0.926
Xie, China, 2020 [27]	Distinguish SCN vs. MCN	· Retrospective · Surgically resected SCN or MCN	2010–2019	57 (26 SCN, 31 MCN)	MDCT	1942	27 features: · 9 Radiological features: lesion size, CT value of cystic fluid, location, lesion shape, cyst wall, enhancement of the wall, calcification, septations, and mural nodules. · 18 high-order radiomic features.	Combined model: radiological + radiomics	Internal	AUC: 0.994 Sn: 96.8% Sp: 100%
Shen, China, 2020 [29]	Discriminate PCL type	· Retrospective · Surgically resected PCL	2014–2019	164 (76 SCN, 48 IPMN, 40 MCN)	MDCT	547	9 features: · 4 Radiological features; serum carbohydrate antigen 19-9, sex, age, and serum carcinoembryonic antigen. · 5 Radiomic features (Histogram_Entropy, Histogram_Skeweness, LLL_GLSZM_GLV, Histogram_Uniformity, HHL_Histogram_Kurtosis)	Random Forest; SVM classifier; ANN model Boruta algorithm	Internal	Accuracy: 79.59% Precision IPMN: 90% Precision MCN: 90% Precision SCN: 72%
Wei, China 2019 [26]	Distinguish SCN vs. other PCLs	· Retrospective · Surgically resected PCL	2007–2016	260 (102 SCN, 74 IPMN, 35 MCN, 49 SPN)	MDCT	409	22 features: 5 Radiological guideline-based: sex, location, shape, and cyst size. 17 Radiomics high- throughput: intensity T-range, wavelet intensity T-median, and wavelet NGTDM busyness.	LASSO SVM	Internal	AUC: 0.84 Sn: 67% Sp: 82%
Distinguish SCN vs. MCN	Distinguish SCN vs. MCN	· Retrospective · Surgically resected SCN or MCN	2013–2018	77 (52SCN, 25 MCN)	MDCT	28	5 Radiomic features: GLRLM_Short-nun high gray-level Emphasis GLRLM_Gray-level non-uniformity GLRLM_Run length non-uniformity GLZLM_Long-zone emphasis GLZLM_Short-zone	LASSO, Random Forest Method	Internal	AUC: 0.77 Sn: 95% Sp: 83%
Dimitriev, USA, 2017 [28]	Discriminate PCL type	· Surgically resected PCL		134 (74 IPMN, 14 MCN, 29 SCN, 17 SPN)	MDCT	11	14 features: · 3 Radiological features: age and gender, cyst location. · 11 Radiomic features: intensity features (mean, STD, Kurtosis, Skewness and median of intensities) and shape features (volume, surface area, surface area to volume ratio, rectangularity, eccentricity and convexity).	Random forest and CNN	Internal	Accuracy: 83.6%

## Data Availability

No new data were created or analyzed in this study. Data sharing is not applicable to this article.

## Abbreviations

The following abbreviations are used in this manuscript:

MRI	Magnetic Resonance Image
CT	Computed Tomography
PCL	Pancreatic cystic lesion
IPMN	Intraductal Papillary Mucinous Neoplasm
MCN	Mucinous Cystic Neoplasm
SCN	Serous Cystic Neoplasm
CT	Computed Tomography
EUS	Endoscopic Ultrasound
CE-EUS	Contrast-Enhanced Endoscopic Ultrasound
ROI	Region of Interest
GLCM	Gray Level Co-occurrence Matrix
GLRLM	Gray Level Run Length Matrix
GLSZM	Gray Level Size Zone Matrix
LASSO	Least Absolute Shrinkage and Selection Operator
AdaBoost	Adaptive Boosting
SVM	Support Vector Machine
AUC	Area Under the Curve
ROC	Receiver Operating Characteristic
DICOM	Digital Imaging and Communication in Medicine
NIFTI	Neuroimaging Informatics Technology Initiative
PACS	Picture Archiving and Communication System
HU	Hounsfield Unit
AP	natomical Pathology
MRCP	Magnetic Resonance Cholangiopancreatography
IQR	Interquartile Range
SD	Standard Deviation
TP	True Positive
TN	True Negative
FP	False Positive
FN	False Negative
